# Apoptotic Potential of Glucomoringin Isothiocyanate (GMG-ITC) Isolated from *Moringa oleifera* Lam Seeds on Human Prostate Cancer Cells (PC-3)

**DOI:** 10.3390/molecules28073214

**Published:** 2023-04-04

**Authors:** Nurul Ashikin Abd Karim, Aziza Hussein Bakheit Adam, Mohammed Sani Jaafaru, Yaya Rukayadi, Ahmad Faizal Abdull Razis

**Affiliations:** 1 UPM-MAKNA Cancer Research Laboratory, Institute of Bioscience, Universiti Putra Malaysia, Serdang 43400, Malaysia; 2 Natural Medicines and Products Research Laboratory, Institute of Bioscience, Universiti Putra Malaysia, Serdang 43400, Malaysia; 3 Department of Food Hygiene and Safety, Faculty of Public and Environmental Health, University of Khartoum, Khartoum 11111, Sudan; 4 Medical Analysis Department, Faculty of Science, Tishk International University, Erbil 44001, Iraq; 5 Department of Food Science, Faculty of Food Science and Technology, Universiti Putra Malaysia, Serdang 43400, Malaysia; 6 Laboratory of Food Safety and Food Integrity, Institute of Tropical Agriculture and Food Security, Universiti Putra Malaysia, Serdang 43400, Malaysia

**Keywords:** glucomoringin, isothiocyanate, apoptosis, prostate cancer

## Abstract

Inhibition of several protein pathways involved in cancer cell regulation is a necessary key in the discovery of cancer chemotherapy. *Moringa oleifera* Lam is often used in traditional medicine for the treatment of various illnesses. The plant contains glucomoringin isothiocyanate (GMG-ITC) with therapeutic potential against various cancer cells. Therefore, GMG-ITC was evaluated for its cytotoxicity against the PC-3 prostate cancer cell line and its potential to induce apoptosis. GMG-ITC inhibited cell proliferation in the PC-3 cell line with IC50 value 3.5 µg/mL. Morphological changes as a result of GMG-ITC-induced apoptosis showed chromatin condensation, nuclear fragmentation, and membrane blebbing. Additionally, Annexin V assay showed proportion of cells in early and late apoptosis upon exposure to GMG-ITC in a time-dependent manner. Moreover, GMG-ITC induced a time-dependent G2/M phase arrest, with reduction of 39.1% in the PC-3 cell line. GMG-ITC also activates apoptotic genes including caspase, tumor suppressor gene (p53), Akt/MAPK, and Bax of the proapoptotic Bcl family. Early apoptosis proteins (JNK, Bad, Bcl2, and p53) were significantly upregulated upon GMG-ITC treatment. It is concluded that apoptosis induction was observed in PC-3 cells treated with GMG-ITC. These phenomena suggest that GMG-ITC from *M. oleifera* seeds could be useful as a future cytotoxic agent against prostate cancer.

## 1. Introduction

Cancer is well known as one of the deadly diseases at present, which can be categorized as a death sentence due its consequences or series of events, and stages that some cancer patients have to go through. Cancer can be described as a disease caused by aberrant cells that grow uncontrollably so that it evades, erodes, then affects or destroys normal cells or tissues. According to the International Agency for Research on Cancer (IARC), there are about 12 common types of cancer that occur among citizens nationwide. Five types of cancers that most commonly occur in males include colorectal, lung, nasopharynx, lymphoma, and prostate cancer [1]. The disease can attack young children and adults, regardless of race; however, men who are more than 50 years old are more at risk of developing prostate cancer.

Due to the increasing number of cancer cases, it is important to discover more potential chemotherapeutic agents against cancer. In cancer treatments, the most common ways to treat cancers are through surgery, chemotherapy, and radiation. Most cancer patients will undergo surgery followed by chemotherapy. Side effects are oftentimes reported by patients undergoing chemotherapy treatment, which include hair loss, fatigue, bruises, and hormonal problems [2]. Therefore, it is crucial to study medicinal plants and their natural compounds to counter the lack of natural anticancer treatment drugs effectively.

Exploration of natural sources in the search for biologically active compounds, which could be applied in anticancer therapy or chemoprevention, has been practiced with success for a long time. Medicinal plants that contain phytochemicals such as alkaloids, polyphenols, and flavonoids provide a significant contribution in reducing cancer. The use of medicinal plants for disease treatment has been reported in the past. Natural plant sources played an important role in discovering alternative medicine that helps prevent cancer. It was reported that around 250,000 plant species are believed to have anti-cancer properties. Medicinal plants are regarded to have a high potential for novel drug discovery and nutraceuticals, and also provide food supplements [3,4].

The discovery of the podophyllotoxin plant compounds led to cancer treatment drugs for testicular and lung cancer [5]. Paclitaxel and docetaxel were discovered from natural plant compounds in the Pacific Yew tree and used in cancer treatments [6]. These compounds were derived from plants and are utilized as natural anti-cancer agents in the market. In fact, ovarian cancer survivors were also found to have early menopause, as well as diminished ovarian function, which are mainly caused by procarbazine and alkylating agents [7].

**Figure 1:**
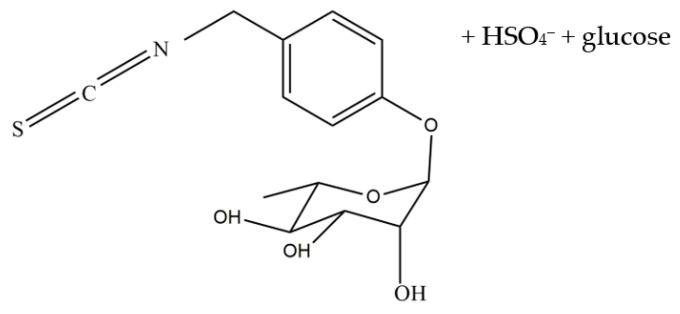
Structural formula of GMG-ITC.

Glucosinolates, which are derived from isothiocyanates (ITC) such as benzyl isothiocyanate (BITC), allyl isothiocyanate (AITC), and phenylethyl isothiocyanate (PEITC), have been reported for their protective effects against cancer cells [8]. The ITCs showed cytotoxic effects towards breast, liver, and lung cancer via modulation of MEK/ERK, PI3K/AKT, and Nrf2 intrinsic and extrinsic apoptosis pathways [8]. However, there has been a lack of study on glucomoringin isothiocyanate (GMG-ITC) (Figure 1) against prostate cancer cell lines to determine its cytotoxic effect. GMG-ITC is a hydrolysis product of a rare glucosinolate known as glucomoringin (GMG) which was isolated from the seed of *Moringa oleifera* Lam [9,10]. GMG-ITC was reported to have arrays of biological activities including anti-inflammatory, anti-oxidant, anti-microbial and anti-ulcer [11]. Isothiocyanates (ITCs) contain anti-cancer agents that may provide the solution for natural anti-cancer treatment with fewer side effects for prostate cancer treatment. This study provides more facts on the cytotoxic potency of glucomoringin isothiocyanate (GMG-ITC) to induce apoptosis in prostate cancer cells and determines the pathways involved to inhibit tumor or cancer progression.

## 2. Results

### 2.1. Crude Extraction and Phytochemical Analysis of M. oleifera Lam Seed

**Figure 2:**
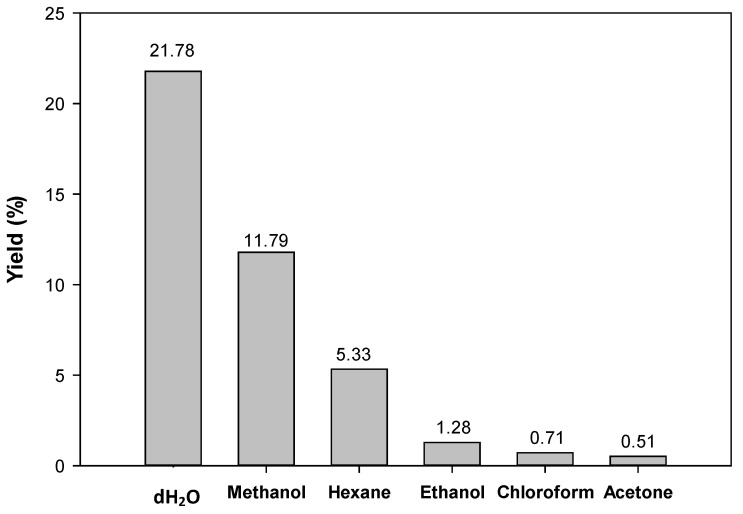
Yield of *M. oleifera* crude extracts.

Crude extraction of *M. oleifera* seeds was performed before the isolation of the compound of interest, and the yield production of crude extract was considered to determine the best solvent to extract the seeds of *M. oleifera*. The highest yield was crude water extract (dH_2_O) (21.78%), followed by methanol (11.79%), hexane (5.33%), ethanol (1.28%), chloroform (0.71%), and acetone (0.51%) as shown in Figure 2.

The findings of this study suggest that distilled water was the best solvent for the extraction of *M. oleifera* seeds because it yields the highest percent of crude extract. Therefore, the crude water extract was chosen for the isolation of glucomoringin to maximize the yield of the compound. The phytochemical analysis of the crude water extracts revealed the presence of several different phytochemicals including saponin, alkaloid, and coumarin.

### 2.2. Characterization of the Isolated Glucomoringin

The yield from the starting material for crude water extract was used for isolation of the compound of interest. The findings of this study suggest that glucomoringin (GMG) was successfully purified from the crude water extract according to the HPLC, NMR, and LCMS data. It was found that the crude extract yielded 9.433% of the GMG compound (Table 1).

The GMG compound was identified and confirmed with NMR and LCMS analysis. The HPLC chromatogram of isolated GMG and the base peak of GMG from the DIMS spectrum are presented in the Supplementary Materials. LCMS analysis confirmed the mass of the compound, which was 588 m/z.

Analysis of ^1^H NMR spectral data shows the presence of a doublet deshielded at 4.74 and multiplet at 2.95 to 3.33, suggesting the presence of glucose moiety. The presence of an aromatic compound was suggested to be deshielded at 7 and 7.21 with doublet, and 4.6 with singlet. The doublet was also deshielded, and suggests a rhamnose moiety, which was at 4.55, 3.85, and 1.16. Multiplet and triplet were detected at 3.68 and 3.3, respectively, and at 5.4 was identified as a broad singlet. The GMG compound was identified as a glucosinolate molecule and was assigned on the basis of spectral similarities to ^1^H and ^13^C NMR. A summary of ^1^H and ^13^C NMR spectral data of GMG is presented in the Supplementary Materials. Furthermore, the isolated GMG was activated to GMG-ITC by a myrosinase enzyme and was subsequently confirmed by HPLC for the cytotoxic studies.

### 2.3. Cytotoxic Effect of GMG-ITC on PC-3 Human Prostate Cancer Cells

**Figure 3:**
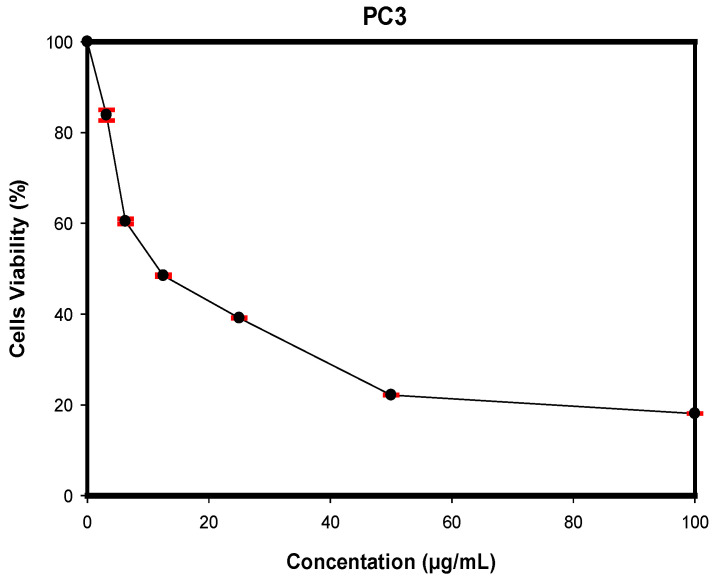
The effects on cell viability upon treatment with GMG-ITC at different concentrations against PC-3 cell line after 72 h of incubation. The data represented mean ± SD of three independent experiments (*n* = 3).

The cytotoxic effect of GMG-ITC derived from the crude water extract of *M. oleifera* Lam seeds was evaluated on the PC-3 cell line with an MTT assay after 72 h exposure (Figure 3). The GMG-ITC showed cytotoxic effect in a concentration-dependent manner with an IC_50_ of 3.5 µg/mL.

### 2.4. Assessment of Apoptosis in PC-3 Cells Induced by GMG-ITC

#### 2.4.1. Morphological Assessment

**Figure 4:**
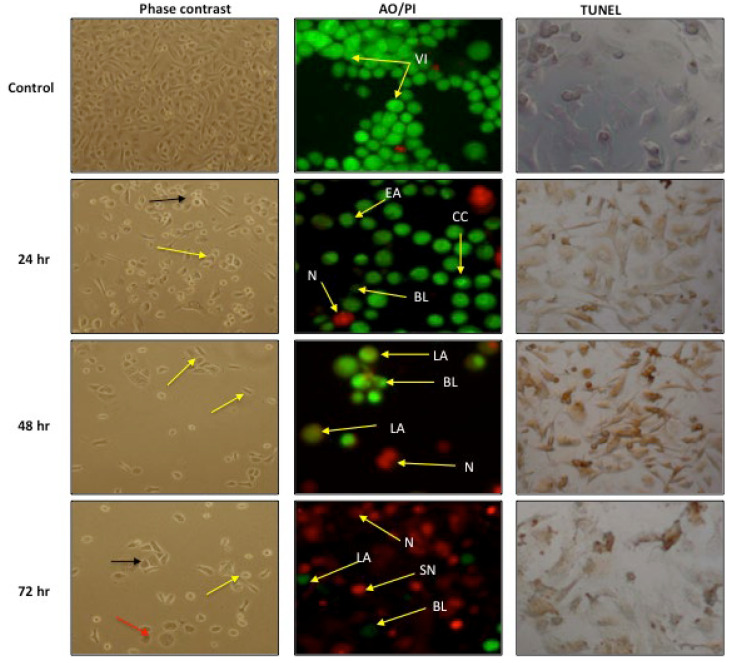
Images of stained PC-3 cells treated with GMG-ITC (3.5 µg/mL) in a time-dependent manner. Phase contrast images of apoptotic PC-3 cells with arrows indicating chromatin condensation in the cell nucleus with occasional enucleation; (black arrow), elongated lamellipodia (yellow arrow), and cells detached from substrate (red arrow). AO/PI fluorescence image of PC-3 cells with arrow indicating (BL) blebbing, (CC) chromatin condensation, (EA) early apoptosis, (LA) late apoptosis, (N) necrosis, (SN) secondary necrosis, and (VI) viable cells. 40× magnifications. TUNEL assay images of PC-3 cells with darkened stains indicating DNA fragmentation within the cells. Magnification 400×.

Induction of apoptosis in PC-3 cells after treatment with GMG-ITC was assessed by a phase contrast microscope after using TUNEL and AO/PI staining methods. The exposed cells clearly show apoptotic features in PC-3 cells including chromatin condensation in the cell nucleus with occasional enucleation, elongated lamellipodia, and cell detachment from their substrate, which became more apparent as the treatment period increased (Figure 4).

In the phase contrast microscopy evaluation, the cells showed clear apoptosis characteristics, as evidenced by their morphology after GMG-ITC treatment. The treated cells showed an increased chromatin condensation in the cell nucleus with occasional enucleation, elongated lamellipodia, and cell detachment from their substrate. Interestingly, these characteristics increased in a time-dependent manner. At 24 h of incubation, the cells comprised less healthy cells compared with untreated cells, and more cells with chromatin condensation and occasional enucleation. At 48 h, more cells were detached, which was a clear sign of unhealthy cells, and the cells were presented with an elongated lamellipodia. At 72 h, there were more cells with apoptosis morphology, which underwent chromatin condensation, elongated lamellipodia, and detachment from their substrate. Apart from that, all treated cells appeared to lose their cell structure with increasing incubation period. The control cells remained intact and were evenly shaped.

#### 2.4.2. Apoptosis Assessment Using Flow Cytometry

**Figure 5:**
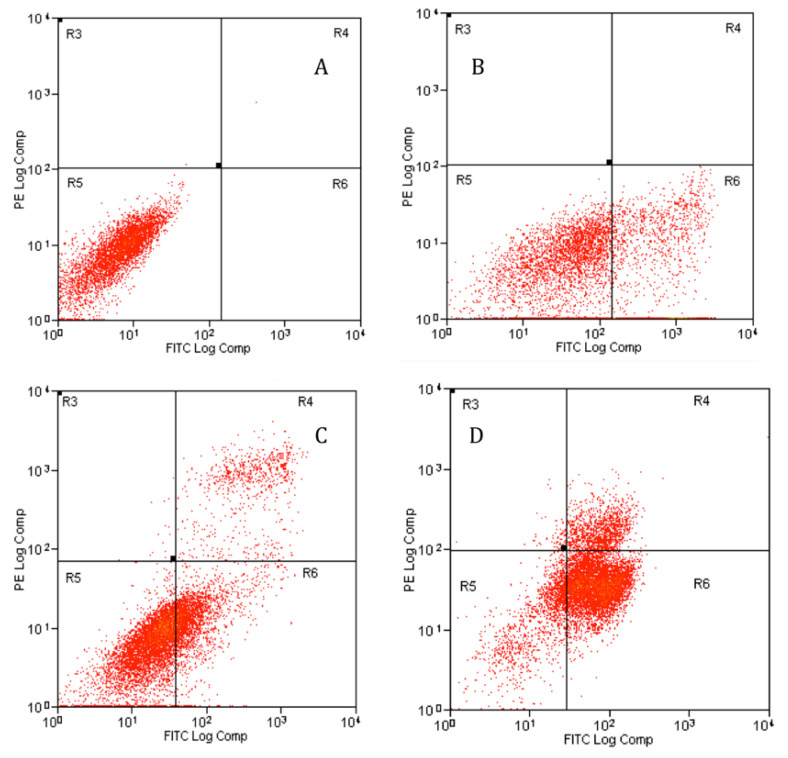
Annexin V-FITC analysis of PC-3 cells treated with GMG-ITC (3.5 µg/mL) for 24 (**B**), 48 (**C**), and 72 (**D**) hours, with (**A**) representing the untreated cells. Early apoptosis (Annexin+/PI−) is shown in the lower right quadrant (Q4) for each panel while late apoptosis (Annexin+/PI+) is shown in the upper right quadrant (Q2). Viable cells are represented in the lower left quadrant (Q3). Necrosis (Annexin−/PI+) is shown in the upper left quadrant (Q1). Results represent one of three independent experiments.

Apoptosis assessment rate was further studied by the Annexin V-FITC staining method using flow cytometry. The Annexin V-FITC plots in Figure 5 shows the PC-3 cell distribution within four different quadrants (Q1, Q2, Q3, and Q4). A uniform induction of apoptosis by GMG-ITC on PC-3 cells was observed in a time-dependent manner from 24 to 72 h (Figure 5). The study revealed that GMG-ITC induced early- and late-stage apoptosis in PC-3 cells as shown in Figure 5. A decrease in viable cells was seen in all the treated cells (Figure 5B–D) compared to the untreated cells (Figure 5A). During the first 24 h, the viable cells decreased from 99.96% to 69.91%. As the treatment duration increased to 48 h, the viable cells’ percentage decreased further to 65.73%, and 16.1% after 72 h.

**Figure 6:**
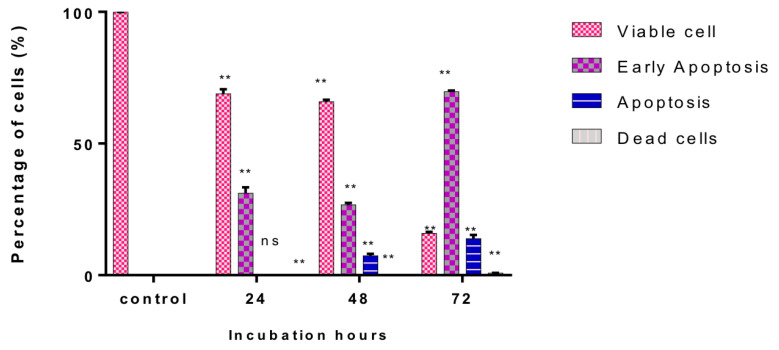
Time dependence of apoptosis rate of exposed PC-3 cell line. Each treatment and control shows the percentage of dead, viable, early apoptosis, and late apoptosis cells. Incubation time for control was the same as treated cell, which were 24 h, 48 h and 72 h. The data represented mean ± standard deviation (SD) of three independent experiments (*n* = 3). Statistical analysis is expressed as ** *p* < 0.001 and ns is non-significant.

Likewise, the results indicated that the apoptosis rate was increasing according to a time-dependent manner (** *p* < 0.001) compared to the control, from 24 until 72 h of cells’ treatment with GMG-ITC (Figure 6). The treated cells possessed a significantly higher apoptosis rate compared to the control (12.62% ± 2.33), to the value of 21.35% ± 0.55 for 24 h, 34.47% ± 0.79 for 48 h, and 72.9% ± 1.2 for 72 h. The number of viable cells decreased as the incubation hours increased due to some of the cells becoming apoptotic. Apoptosis rate was the highest at the longest incubation period, which suggested that GMG-ITC induction of apoptotic cells was a time-dependent occurrence.

**Figure 7:**
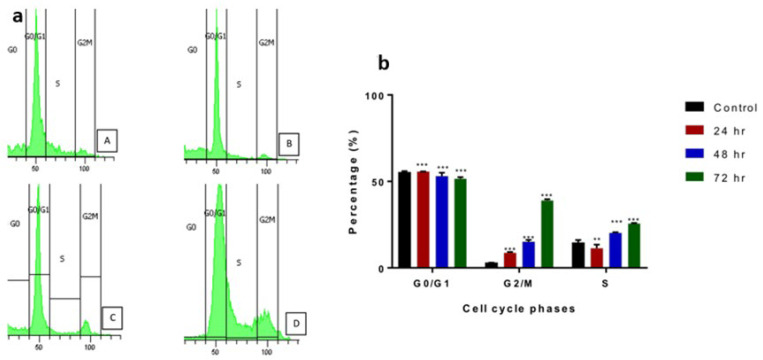
(**a**) represents the proportions of PC-3 cells upon treatment with GMG-ITC based on a time-dependent manner. The proportions of 24 h (B), 48 h (C), and 72 h (D) compared with untreated cells (A), which acts as the control in this experiment. (**b**) represents cell cycle analyses for 24, 48, and 72 h. Data are expressed as *** *p* < 0.0001, ** *p* < 0.005 when compared with the control.

The effect of GMG-ITC on prostate cancer cell cycle phases was also determined. DNA content was measured by flow cytometry. Cell fraction of the treated cells was decreasing at S, followed by the G2/M phase at 24, 48, and 72 h of treatment compared to the control (Figure 7a,b). The G2/M phase started at 3% for control, followed by 8.9% at 24 h, 14.5% at 48 hr and finally 39.1% at 72 h of GMG-ITC exposure. The cell cycle was arrested at the G2/M phase in which the cells’ percentage was at first low at sub G0, followed by a sudden increase at G2/M. The trends were also the same for treatments at 24, 48, and 72 h of incubation of the cells treated with GMG-ITC.

#### 2.4.3. Apoptotic Gene and Protein Expression Modulated by GMG-ITC

**Figure 8:**
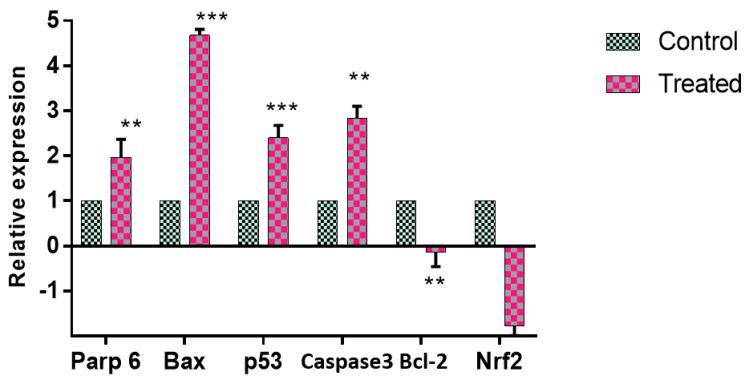
The effect of GMG-ITC on mRNA expression. Values represent fold changes between control and treatment groups. Data are expressed as mean ± SD; ns: non-significant compared to the control, ** *p* < 0.05; *** *p* < 0.001.

GMG-ITC treatment induced expression of certain apoptotic genes in cell signaling. mRNA expressions of p53, Nrf2, Bax, Bcl-2, caspase-3, and parp 6 in PC-3 cells exposed to GMG-ITC were determined by using qPCR (Figure 8). The Nrf2 mRNA expression was significantly decreased in GMG-ITC treatment, while the p53 expression was significantly increased. GMG-ITC significantly upregulated Bax, caspase 3, p53, and Parp 6 and downregulated Nrf2 and Bcl-2 expression. Expression of Bax, at 4.6-fold, was followed by Parp6, higher compared to the control by 1.9-fold. Expression of p53 was significantly higher than the control by 2.135-fold, as well as caspase 3 by 2.69-fold compared to the control. Nrf2 and Bcl-2 were both downregulated compared to the control, with Nrf2 at 0.00196 and Bcl-2 at 0.0071. p53, caspase 3, and parp 6 were pro-apoptotic proteins.

**Figure 9:**
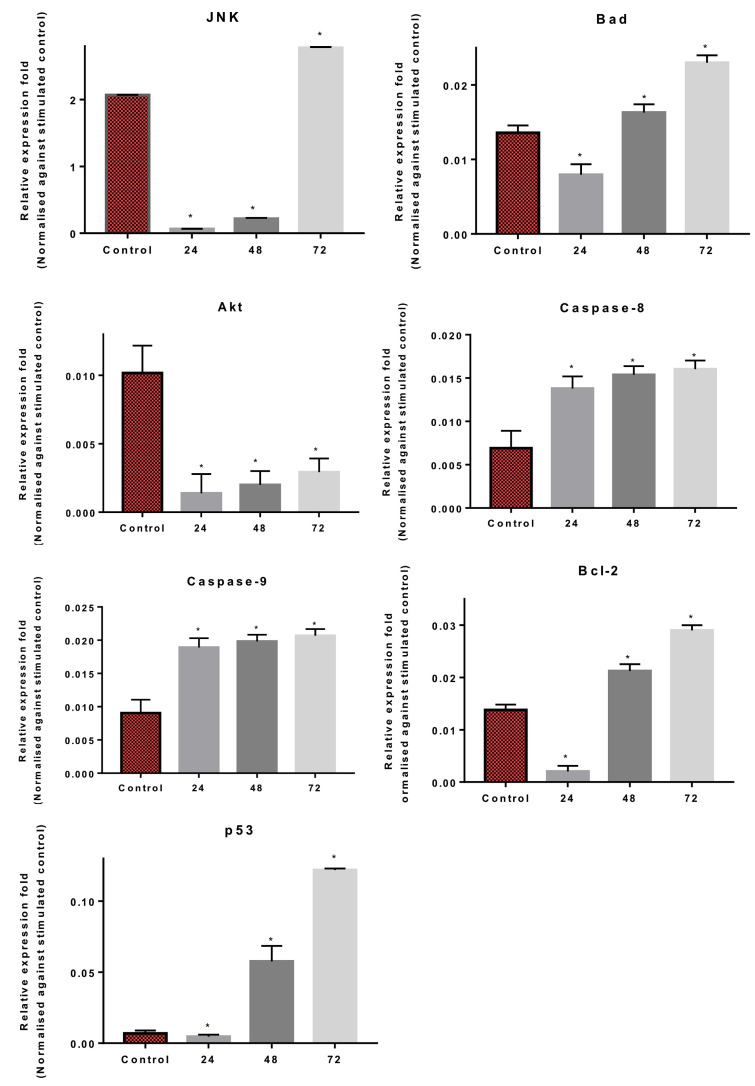
Expression level of apoptosis-related proteins in PC-3 cells treated with GMG-ITC as determined by multiplex analysis. The data are represented as relative expression of proteins in mean ± SD of at least three replicates from three independent tests. An asterisk * indicates statistically significant different from the untreated control (*p* < 0.05).

The cell signaling assay was carried out with a multiplex analysis. The results indicated a detection of early apoptosis via protein expressions in the cell signaling. Early apoptosis activity was observed to occur more when the cells were treated for 24, 48 and 72 h with GMG-ITC (Figure 9). The proteins determined were JNK, Bad, Bcl-2, Akt, Caspases-8 and -9, and p53, which are involved in the apoptosis pathway. The results indicated that GMG-ITC significantly upregulated the expression of p53 and Caspases-8 and 9 in the treated PC-3 cells. However, Akt was significantly downregulated in the treatment. JNK expression was only upregulated when the treatment was at 72 h but significantly downregulated at 24 and 48 h. Bad and Bcl-2 expression were significantly upregulated at 48 and 72 h of treatment but downregulated at 24 h.

## 3. Discussion

The apoptosis pathway has a crucial role in cancer existence and development of drug resistance against a number of anticancer drugs. Currently, it is an urgent need of the present time to understand the role and mechanism of apoptosis-related proteins in cancer. Use of medicinal plants as an approach in the prevention and treatment of cancer has been followed for many years and many therapeutic plants with anticancer activity are reported in the literature [12,13]. In addition, adverse effects and drug interactions are major restrictions in synthetic anticancer drugs; therefore, plants have been investigated across the world to exploit novel and potential sources of anticancer agents.

*Moringa oleifera* Lam is often used in traditional medicine in several countries for the treatment of various illnesses. The plant was also found to contain bioactive compounds with therapeutic potential against various cancer cells [14]. This study was carried out to determine the apoptotic effect of isolated glucomoringin (GMG) from the seeds of *M. oleifera* on prostate cancer cell lines.

The findings of this study suggest that distilled water was the best solvent for the extraction of *M. oleifera* seeds because it yields the highest percent (21.78%) of crude extract. Therefore, the crude water extract was chosen for the isolation of glucomoringin to maximize the yield of the compound. Previous studies have reported the crude water extract yielded the highest compared to other solvents [15,16]. Crude water extract has been used extensively in various traditional medicines due to its minimal side effects compared to organic solvents [16,17,18]. In addition, an extraction using crude water has been reported to inactivate enzymes in the plant itself, and enables the extraction of water soluble-compounds in high amounts [19]. Phytochemical analysis conducted on crude water extract revealed the presence of constituents that are known to exhibit medicinal as well as physiological activities [20]. Analysis of the crude water extract revealed the presence of phytochemicals such as saponin, alkaloid, and coumarin. Interestingly, alkaloids have been associated with medicinal uses for centuries and one of their common biological properties is their cytotoxicity against cancer cells [21,22]. Moreover, alkaloids in plants generally have potential as anti-cancer agents because they were reported to inhibit the topoisomerase enzyme, which is essential during DNA replication, apoptosis induction, and upregulation of p53 gene expression [23,24,25]. It was also reported that alkaloids from *M. oleifera*, the moringins, help to relax the bronchioles [26].

The findings of this study suggest that glucomoringin (GMG) was successfully isolated and purified from the crude water extract according to the HPLC, NMR, and LCMS data (presented in Supplementary Materials), which are consistently similar to data established previously [27,28,29]. The isolated GMG was activated to GMG-ITC by the myrosinase enzyme and was subsequently confirmed by HPLC as suggested by a previous study [28].

The cytotoxic effect of the isolated compound (GMG-ITC) derived from the crude water extract of *M. oleifera* Lam seeds was evaluated on PC-3 human prostate cancer cells with an MTT assay during 72 h. The GMG-ITC showed cytotoxic effect on PC-3 cells with an IC_50_ value at 3.5 µg/mL (Figure 2). The low range of IC50 values is preferable according to the US National Cancer Society guidance for pure compounds (less than 4 µg/mL) [30]. The observation of the cytotoxic and apoptotic potential of the compound was further examined by other techniques.

The cells undergoing apoptosis have a series of morphological apoptotic specifications such as cell shrinkage, membrane blebbing, chromatin cleavage, nuclear condensation, and apoptotic body formation [31]. Therefore, morphological apoptotic changes in PC-3 cells exposed to GMG-ITC were assessed.

Our data in this study indicate variable morphological apoptotic changes including chromatin condensation in the cell nucleus with occasional enucleation, elongated lamellipodia, and cell detachment from their substrate. Furthermore, it is interesting to note that the morphology of GMG-ITC-treated cells showed distinct apoptosis characteristics in a surprisingly time-dependent manner, which became more apparent as the treatment period increased. Consistently, previous studies showed condensed and fragmented nuclei in cells when exposed to GMG-ITC [32]. It has been reported that apoptotic chromatin condensation is the direct result of DNA fragmentation in apoptosis [33,34].

Additionally, apoptosis assessment rate was further studied by the Annexin V-FITC staining method using flow cytometry. In Annexin V-FITC, the apoptosis cell activity increased significantly in a time-dependent manner. The treatment with GMG-ITC on PC-3 cells demonstrated that it significantly reduced the mitochondrial transmembrane potential with attainable concentrations, was able to induce the release of cytochrome C, and finally increased annexin v binding. According to Hantz et al. [35], these characteristics confirmed apoptosis induction in the cells.

Likewise, GMG-ITC was also observed to arrest the cell cycle at the G2/M phase, which is an important phase in apoptosis. Cell cycle analysis by flow cytometry is a widely used procedure to analyze cell cycle arrest in cancer. An effective chemoprevention strategy relies on agents that can selectively eliminate proliferating cells via programmed cell death and spare quiescent or terminally differentiated cells [36]. AKT and MAPK pathways were activated when the cell cycle was arrested at the G2/M phase, thus contributing to apoptosis [37]. This finding can be considered to be a hallmark of apoptotic cell death. These results suggest that GMG-ITC is capable of selectively inhibiting cellular proliferation and accelerating apoptotic events in prostate cancer cells. It also offers crucial promise as a chemo-preventive or therapeutic agent against prostate cancer cells. Cell cycle arrest at the G2/M phase is far more important than at G0/G1, however, as the apoptosis percentage was increasing, suggesting that other pathways may be involved [38].

To further explore the molecular events involved in apoptosis, gene expression and protein analysis has been carried out. Quantification of mRNA indicates Nrf2 expression was downregulated significantly, while p53, Bax, and parp 6 were upregulated by the treatment with GMG-ITC against PC-3 cell lines. The expression of p53 can be linked with a blockage of cell cycle progression at the G2/M phase and the upregulation of Bax and caspase-3, while Bcl2 and Nrf2 were downregulated. The p53 pathway has been shown to mediate cellular stress responses; p53 can initiate apoptosis [39]. Moreover, many of the genes regulated by p53 have been shown to participate in apoptotic pathways (including Bax, parp, and Bcl2); however, many features of the mechanism underlying p53-mediated apoptosis remain unresolved [40]. In contrast, increase in expression of Bax is well known to induce cell death, eliminating tumor cells [40]. It has been suggested that a high ratio of Bax to Bcl2 can lead to collapse of mitochondrial membrane potential, resulting in release of cytochrome c and consequently causing cell apoptosis [41]. Our data also confirm that decreased Bcl2 protein expression removes its inhibitory effect on Bax and caspase, and leads to over expression of Bax and finally activation of caspase.

Finally, protein analysis employing cell signaling immunoassay studies by multiplex analysis revealed the modulation of apoptotic proteins after the treatment of the cancer cells with GMG-ITC. The expression of proteins JNK, Bad, Bcl2, caspase, and p53 in the analysis was significantly upregulated compared to control. The expression is consistent with the multiplex analysis whereby the assay confirms early apoptotic activity in the treated cells with the expression of JNK, Bad, Bcl-2, caspase-8, caspase-9, and p53 proteins. However, in contrast with mRNA quantification, the compound did not alter the expressions of caspases-7, but upregulated caspase -8 and -9, which determined the apoptosis induced by GMG-ITC in PC-3 cells is also caspase-mediated. The activated isothiocyanate was found to induce apoptosis via a caspase-mediated pathway in treated breast cancer and nude mice in vivo [27]. The determination of apoptosis is not exclusively by caspase activation because execution of apoptosis can occur in the absence of caspases [42]. The execution can be also facilitated by other non-caspase proteases such as endonuclease and cathepsin. However, other important hallmarks for cell shrinkage and detachment of substrate can occur in caspase-dependent apoptosis. Moreover, sequential activation of caspases plays an important key role in the execution phase of cell apoptosis [43]. Activation of caspase in the protein study determined that GMG-ITC induced caspase activity in PC-3-treated cells.

Glucomoringin isothiocyanate is highly soluble in water due to the structure of the compound, which is high polarity. It is also known as a hydrolysis product of glucosinolates. According to a previous study, high polarity of the compound makes it easily dissolved in water and exerts various anti-tumor activities [44]. According to Jed et al. [45], though low concentration of glucosinolates is present in vacuoles of most tissues of the plant, the level can be as high as 26% by weight in the seeds. Dietary glucosinolates are absorbed by humans after they are converted to isothiocyanates in the presence of naturally occuring enzymes and microflora in the stomach, which can be further metabolized in humans [46]. Thus, this explains its bioactivity or pharmacokinetic properties against prostate cancer or other illness. The metabolites can also be observed by using the high-resolution, accurate mass-mass spectrometry (HRAM-MS) method to further investigate their existence in human urine. A study by Jianghao et al. [47] indeed revealed the targeted metabolite analysis of glucosinolates in human urine of six subjects. The metabolites were also found in the plasma after 2 h of consuming, which served as marker compounds for the intake of converted glucosinolates.

In many cell and animal models, there have been various studies showing consistency of glucosinolates against prostate cancer. According to Tracey et al. [48], there are three possibles ways of glucosinolates distribution in the prostate gland. First, it may be through hepatic metabolism which results in expression of NF-E2–related factor 2 (‘Nrf2’), which maintains the positive effect of healthy prostate gland tissues. The second is to change gene expression and metabolism, which reduces risk of prostate cancer. Finally, the metabolites may accumulate in prostate tissue or through urinary reflux which may accumulate to a certain amount or concentration that maintains a healthy prostate. An in vivo study by Chu et al. [49] indicates that glucosinolates can accumulate in the prostate gland of a mouse model; however, there is currently no evidence of its accumulation in human studies. So, it can be concluded that there is possible distribution or accumulation of glucosinolates in the urinary tract and prostate if we can refer to previous studies.

## 4. Materials and Methods

### 4.1. Plant Materials

The *Moringa oleifera* Lam seeds used in this study were purchased from a local company, The Mitomasa Sdn. Bhd. The seeds were harvested from Sarawak. Taxonomic identification was carried out at the Biodiversity Unit, Institute of Bioscience, Universiti Putra Malaysia.

### 4.2. Crude Extractions

Extraction of *M. oleifera* seeds in various solvents was performed to identify the most active crude against cancer cells following a modified method by Tiloke et al. [50]. The dried seeds were ground into powder using a grinder (Retsch grinder Model ZM1, Haan, Germany). Ten grams of dried powder were soaked into 100 mL of different solvents separately for 24 h in room temperature, including ultrapure water, ethanol (Sigma, St. Louis, MO, USA), methanol (Sigma), acetone (Sigma), chloroform (Sigma), and hexane (Sigma). The solvents were then filtered using filter paper, and the volume reduced by 9 times using a rotary evaporator. The extraction was repeated using the same method and the final extract was weighed and stored at 4 °C until further use.

### 4.3. Phytochemical Analysis

Qualitative phytochemical screenings of the six different solvents of the crude extracts obtained from crude extraction were performed. The tests were carried out using standard procedures described by Monika et al. [51] and Hossain et al. [17]. The screenings for saponin, alkaloid, quinone, coumarin, and fixed oil were carried out as well.

### 4.4. Extraction and Purification of Glucomoringin

Extraction of glucomoringin (GMG) from the seeds of *M. oleifera* was done according to the method described by Visentin et al. [52]. Five hundred grams of the seeds were oven-dried for 48 h at 30–40 °C. The dried seeds were ground in a grinder (Retsch grinder Model ZM1, Haan, Germany) equipped with a 0.75 mm sieve. Dry powder was obtained and stored in a sealed vessel at room temperature until further use. Fifty grams of plant powder was added to ultrapure water in the ratio of 1:30 (w/v). The sample was extracted twice with boiling water and then homogenized with an Ultraturrax Model TP 18/2N (IKA, Kuala Lumpur, Malaysia) for 15 min at medium speed, followed by centrifugation at 17,700× *g* for 30 min using a Beckman Model J-21 (Beckman Coulter, Indianapolis, IN, USA).

Purification of the crude extract was performed according to a previous method described by Barillari et al. [53]. One molar of zinc acetate (Zn (OAc)2) was added into the supernatant in the ratio of 1 to 50 (*v*/*v*), and the mixture was centrifuged again at 1000× *g* to remove any precipitated protein. The deproteinized extract was loaded on a DEAE-Sephadex A-25 (Pharmacia, Stockholm, Sweden) anion-exchange column (26 × 150 mm) with 25 mM acetate buffer at pH 5.6, and the column was washed with 1 L of distilled water. The elution was carried out with 500 mL of 0.1 M potassium sulphate (K_2_SO_4_). A rotary evaporator was used to concentrate the eluted solution to dryness at 70 °C under vacuum. Three subsequent extractions were carried out with 70 mL of boiling methanol (MeOH). The alcoholic extracts were filtered and concentrated to approximately 15 to 20% of the initial volume. The solution was warmed and slowly added drop by drop to 200 mL of ethanol (EtOH), which was previously cooled to 20 °C until a white powder precipitation was obtained. The solution was centrifuged and solid glucomoringin was obtained (potassium salt) and freeze-dried. It was then sealed under vacuum condition to prevent any moisture uptake due to its highly light-sensitive nature. The white powder was further analyzed by HPLC.

### 4.5. HPLC Analysis of Glucomoringin

The compound analysis was carried out using a Hewlett-Packard Model 1100 HPLC (HPLC) system with an Inertsil ODS3 column (250 × 3 mm, 5 µm). The purity of the compound was assayed according to the European Economic community official method (ISO 9167-1), based on the HPLC analysis of the desulfo-glucosinolates obtained through the removal of the sulfate group by sulfatase-catalyzed hydrolysis [53]. The yield of the glucomoringin (GMG) was of 99% purity based on peak area value. It was about 90–92% on a weight basis, which was due to its highly light-sensitive properties. The chromatography was performed with 1 mL/min flow rate at 30 °C by eluting with a gradient of water as the A solution, and acetonitrile as the B solution as follows: isocratically 1% B for one-minute, linear gradient to 22% B for 21 min, and linear gradient to 1% B for three minutes. A diode array detected the desulfo-glucomoringin by monitoring the absorbance at 229.5 nm and the response factor of 1.1 referred to desulfo-sinigrin as the standard. The white powder was then analyzed by 1H- and 13C-NMR spectrometry.

### 4.6. NMR Analysis of Glucomoringin

The compound was further analyzed by 1H- and 13C-NMR spectrometry. Optical rotation was measured at 20 °C using a Perkin-Elmer 410 polarimeter. The mass spectra were obtained using the ion spray technique with an API 300 Perkin-Elmer spectrometer (SCIEX, Framingham, MA, USA). ^1^H and ^13^C-NMR spectra were recorded in D_2_O solution on an AMX500 spectrometer (Bruker, Billerica, MA, USA) operating at 500 and 125.7 MHz, respectively.

### 4.7. Bioactivation of Glucomoringin

The enzyme myrosinase (MYR) (Sigma, USA) was used to hydrolyze the glucomoringin to glucomoringin isothiocyanate (GMG-ITC). The stock solution of MYR used in the present study had a specific activity of 60 units/mg of soluble protein and was kept at 4 °C after dilution in H_2_O at 34 U/mL. One MYR unit was defined as the amount of enzyme able to hydrolyze 1 mmol/min of sinigrin at pH 6.5 and 37 °C [54]. The GMG was dissolved in distilled water at a concentration of 1 mM as a stock solution, and kept at 20 °C. The solutions were diluted in a non-serum media (RPMI) with 0.1 M phosphate buffer (pH 6.5) at 37 °C at the desired concentrations. In every treatment, 5 µL of MYR was added for every 1.5 mL of GMG solutions in order to produce the active ITCs. The total conversion of pure GMG into GMG-ITC was confirmed by HPLC analysis of the desulfo-derivative (EEC Regulation No. 1864/90 Enclosure VIII. Offic. Eur. Commun. L170: 27-34), which allowed us to monitor the reduction until complete disappearance of GMG in the reaction mixture. Acetonitrile was then added to the mixture of MYR and GMG until the final concentration was 20% and GMG-ITC was purified by reverse phase chromatography, which was performed using an HR 16/10 column packed with Li Chrospher RP-C18 (MERCK, New Jersey, NJ, USA), connected to a GradiFrac System (Pharmacia, Germany). After washing with 20% acetonitrile, elution was carried out with a gradient of up to 60% acetonitrile.

Fractions were collected and analyzed using a Hewlett-Packard Model 1100 HPLC (Agilent, Hampton, NH, USA) system with an Inertsil ODS3 column (250 mm × 3 mm, 5 mm). Chromatography was performed with 1 mL/min flow rate at 30 °C by eluting with a linear gradient of water (A) and acetonitrile (B) from 30% B to 80% in 20 min. Elution of GMG-ITC was detected by a diode array, monitoring the absorbance at 229 nm. Fractions containing GMG-ITC (peak purity > 99%) were collected, the solvents were removed by concentration in a rotary evaporator, and the final solution was freeze-dried. The GMG-ITC was characterized and unambiguously identified by ^1^H and ^13^C NMR and mass spectrometry.

### 4.8. Cytotoxicity Study of GMG-ITC

The cytotoxicity test was performed using MTT assay to determine the lowest concentration of GMG-ITC that can inhibit the growth of PC-3 cells. The MTT assay was carried out according to a method as described by Abdelwahab et al. [55]. The cells were cultured and maintained using the RPMI-1640 culture media (PAA, Munich, Germany) until it reached 80–90% confluency. Cells were trypsinized and counted using a hemocytometer, and 1 × 10^4^ cells/mL were seeded in a micro titer plate of 96 wells. The cells were incubated overnight to allow for cells’ attachment. The media was changed, and 0.1 mL of new supplemented media was added into each well. Cells were then treated with GMG-ITC at concentrations of 0, 3.125, 6.25, 12.5, 25, 50, and 100 µg/mL. A 0.1% DMSO was used as negative control and cisplatin was used as a positive control. The cells were subsequently incubated at 37 °C within a humidified chamber with 5% CO_2_ for 24, 48, and 72 h. Negative and positive controls were also incubated for 24, 48 and 72 h. Once the incubation was complete, 20 µL of MTT dye (15 mg/mL) was added into each well, and then the treated cells were incubated again for four hours in dark conditions. After the incubation, 100 µL of DMSO was added into each well and the absorbance reading was performed using an ELISA plate reader (TECAN, SunriseTM, Mannedorf, Switzerland) at 520 nm. The results were expressed as percentage of cell viability divided by control after exposure of the cells to the compounds for 24, 48, and 72 h. The potency of cell growth inhibition of the compound was expressed as IC_50_ values.

### 4.9. Morphological Assessment of Apoptotic Cell Induction

#### 4.9.1. Phase-Contrast Inverted Microscope Evaluation

Morphological changes in the PC-3 cell after treatments with GMG-ITC at 3.5 µg/mL (IC_50_) were observed under an inverted microscope at 24, 48, and 72 h consecutively. In brief, the old media was removed from the 25 mL culture flask (T25) (TPP, Long Island, NY, USA) at the end of the incubation time. The cells were washed once with phosphate buffer saline (PBS) at pH 7.4 and observed under a Leica DMI 3000B phase-contrast inverted microscope (Leica Microsystems, Wetzlar, Germany) at 200× magnifications.

#### 4.9.2. Acridine Orange (AO) and Propidium Iodide (PI) Double Staining

GMG-induced cell death in the treated cells was quantified using the acridine orange (AO) and propidium iodide (PI) double-staining protocol described by Arbab et al. [56] and was examined under a fluorescence microscope (Leica attached with Q-Floro Software, Forchheim, Germany). Briefly, cell treatment was carried out in a 25 mL culture flask (T25). The cells were seeded in the flask at 1 × 10^6^ cells/mL and treated with 3.5 µg/mL of GMG-ITC for 24, 48, and 72 h consecutively. The cells were then centrifuged at 300× *g* for 10 min (Rotofix 32, Hettich Instruments, Beverly, MA, USA). The supernatant was discarded, and the cells were washed twice using PBS buffer after centrifuging at 300× *g* for 10 min to remove the remaining media. Ten microliters of fluorescent dyes containing AO (10 mg/mL) (Sigma-Aldrich) and PI (Sigma-Aldrich) (10 mg/mL) were added into the cellular pellet at equal volumes. A freshly stained cell suspension was dropped onto a glass slide and covered with a coverslip. Slides were then observed under a UV-fluorescence microscope (Nikon, Tokyo, Japan) within 30 min before the fluorescence faded. The percentages of viable, early apoptotic, late apoptosis, and secondary necrotic cells were determined in 200 cells. The criteria for identification are as follows: (i) viable cells appear to have green nucleus with intact structure; (ii) early apoptosis exhibits a bright-green nucleus showing condensation of chromatin in the nucleus; (iii) dense orange areas of chromatin condensation showing late apoptosis; and (iv) orange intact nucleus depicting secondary necrosis [28]. This assay provides a useful quantitative evaluation of apoptosis and was performed in triplicates (*n* = 3).

#### 4.9.3. Dead End Calorimetric Tunel Assay

Firstly, 1 × 10^6^ cells/mL of PC-3 cells were seeded in a T25 culture flask (TPP) and incubated overnight in a humidified chamber. After 24 h, the cells were treated with 3.5 µg/mL of GMG-ITC, and were incubated for 24, 48, and 72 h in separate culture flasks and harvested at the end of each incubation period. The treated cells were then grown onto poly-L-lysine slides overnight. After overnight incubation, the slides were immersed in 4% paraformaldehyde for 25 min, followed by immersion in PBS, twice (for five minutes each). The slides were then immersed in 0.2% Triton^®^ X-100 in PBS for five minutes and washed again twice with PBS for a duration of five minutes each. A 100 µL equilibration buffer was added on the surface of the slides and equilibrated at room temperature for 5–10 min. Subsequently, 100 µL of TdT reaction mix was added onto the cells and the slides were allowed to be semi-dried.

The slides were then covered with a plastic coverslip to make sure the mixture was evenly distributed, followed by incubation for 60 min at 37 °C in a humidified chamber. After 60 min, the plastic coverslips were removed, and the slides were soaked in 2× SSC for 15 min. After that, the slides were washed again three times with PBS (5 five minutes each). The slides were then immersed in 0.3% hydrogen peroxide for 3–5 min and washed three times with PBS (five minutes each). Next, 100 µL of Streptavidin HRP (1:500 in PBS) was added to the slides and the slides were left at room temperature for 30 min. The slides were washed again with PBS three times (five minutes each). Subsequently, 100 µL of DAB, which was freshly prepared prior to use, was added onto the slides until a light background appeared on the slides. The reaction was stopped by immersing the slides a few times in deionized water. Finally, the slide was mounted with permanent mounting medium and the staining of the cells was observed under a light microscope (Nikon).

### 4.10. Cell Cycle Analysis by Flow Cytometry

#### 4.10.1. Annexin V-FITC by Flow Cytometry

The procedure was performed following the protocol provided by the manufacturer using the Annexin V-FITC Kit (Sigma-Aldrich, MO, USA). Briefly, 1 × 106 cells/mL of PC-3 cells were seeded in a T25 culture flask and incubated. After the 24 h incubation, the cells were treated with 3.5 µg/mL GMG-ITC and incubated for 24, 48, and 72 h. Later, the cells were collected and centrifuged at 1500× *g* rpm for five minutes. The supernatant was discarded, and the cell pellets were washed once with PBS and put on ice all the time. Then the cells were stained with FITC-Annexin V and analyzed using a flow cytometer (Becton Dickinson, Franklin Lakes, NJ, USA).

#### 4.10.2. Cell Cycle Arrest Analysis by Flow Cytometry

The cell cycle phase arrest was determined by flow cytometry according to the protocol provided by the manufacturer, using the BD cell cycle kit (Pharmingent, London, UK). Briefly, PC-3 cells were harvested at 1 × 10^6^ cells/mL and the cell suspension was transferred into a 15 mL falcon tube and centrifuged at room temperature at 1500× *g* for five minutes.

The supernatant was discarded carefully, and 1 mL of buffer solution was added. The cells were vortexed at low speed and the number was adjusted to 1 × 10^6^ cells/mL by using a buffer solution. Subsequently, trypsin buffer was added to the cells and incubated for 10 min in the dark. Two hundred microliters of solution B (trypsin inhibitor and RNase buffer) was added into the mixture. The cells were tapped slowly to allow mixing and left at room temperature for 10 min. Finally, the cells were then stained with 200 µL of cold solution C (propidium iodide stain solution). The solution was mixed and left at room temperature for 10 min in the dark. The cells were strained using a cell strainer, transferred into a 17 × 100-mm tube and gently tapped. The cells were immediately analyzed using the flow cytometer (BD Accuri, New Jersey, NJ, USA) by analyzing at least 10,000 cells per sample. The percentage of cells in the G1, S, and G2 phases was analyzed by the ModFit LT software 5.0 (Verity Software House, Topshom, ME, USA).

### 4.11. Quantitative Polymerase Chain Reaction (qPCR) for Gene Expression Analysis

Quantification of mRNA was performed to determine the expression levels of the mRNA that are responsible for apoptosis. RNA was isolated from the treated (for 72 h) and the non-treated PC-3 cells using the RNeasy kit (Qiagen, Germantown, MD, USA) as per the manufacturer’s guideline. Spectrophotometric analysis was performed using a Nanodrop (ND-2000) to quantify and ensure the quality of the RNA. The RNA was reverse transcribed to complementary DNA (cDNA) using a Quantinova Extraction Kit (Qiagen) following the manufacturer’s instructions. RT2 SYBR^®^ Green qPCR Master Mix (Qiagen) and a Corbett Rotor-Gene 6000 thermocycler, together with analytical software V 17 (Qiagen Llc, MD, USA), were used to perform quantitative real-time polymerase chain reaction (qPCR). Expression of GAPDH (housekeeping gene), Parp 6, Bax, p53, Caspase 3, BCl-2, and Nrf 2 were quantified by RT-qPCR using Rotor-Gene SYBR^®^ Green Kit. The primers were synthesized by 1st Base DNA (Selangor, Malaysia). Both reverse and forward primers were diluted with 1 × Tris-EDTA buffer to the working concentration (100 nM) and stored at −30 °C until used.

### 4.12. Apoptotic Protein Detection by Cell Signaling Immunoassay

PC-3 cells were treated with 3.5 µg/mL GMG-ITC for 72 h and harvested for the cell signaling immunoassay using a Luminex^®^ xMAP^®^ technology-based MILLIPLEX MAP cell signaling MAPmates™ multiplex assay kit (Merck Millipore, Burlington, MA, USA). The cells were lysed on ice using the lysis buffer from the kit. Particulate matter was removed by filtration and the protein concentration was determined and quantified by BCA protein quantification assay. Then the lysates were mixed with MILLIPLEX buffer, which contains MILLIPLEX map capture beads. The beads comprised target analytes and were incubated overnight at 4 °C. The beads were further incubated with MILLIPLEX MAP DETECTION antibodies after removing the supernatant and washed for one hour at room temperature in the dark. Subsequently, the beads were re-suspended in MILLIPLEX assay buffer and immediately analyzed using LUMINEX 200™ (Luminex Corp, Austin, TX, USA). Nonstimulated PC-3 cells were used as the negative cell lysate control, while EGF-stimulated A431 cell lysates (Millipore, Burlington, MA, USA) were used as the positive cell lysate control and the results were analyzed using xPONENT^®^ software 3.1 Rev 2.

### 4.13. Statistical Analysis

Data were presented as mean ± standard deviation. Significant differences were determined by using one-way ANOVA followed by Tukey’s test, where * *p* < 0.05 denoted a statistically significant difference.

## 5. Conclusions

In conclusion, the study revealed the evidence that GMG-ITC shows cytotoxicity potency through the induction of apoptosis and cell cycle arrest in PC-3 cells. The findings demonstrate that PC-3 cells treated with GMG-ITC arrest at the G2/M phase and that apoptosis is induced through both caspase- and mitochondrial-mediated pathways. GMG-ITC has the potential to promote strong anti-cancer activity against prostate cancer through various mechanisms, and further studies are required to understand the use of the compound in clinical treatment.

## Figures and Tables

**Table 1:** Yield of the isolated compound, glucomoringin.

Initial Weight of Sample (g)	Yield of Crude Water Extract Per 1 g of Sample		Yield of Compound (mg/g) of Crude	
10	g	%	mg	%
	2.17 ± 0.13	21.78	94.33 ± 0.01	9.43

## Data Availability

Data are contained within the article.

## Supplementary Materials

The following supporting information can be downloaded at: https://www.mdpi.com/article/10.3390/molecules28073214/s1, Figure S1: HPLC chromatogram of glucomoringin; Figure S2: HPLC chromatogram of crude water extract of *M. oleifera* seed; Table S1: NMR data of glucomoringin; Figure S3: Mass spectrogram (ESI positive mode) of isolated glucomoringin.

## Sample Availability

Samples of the compounds are available from the authors.
