# Supplementary material for: Apoptotic Potential of Glucomoringin Isothiocyanate (GMG-ITC) Isolated from Moringa oleifera Lam Seeds on Human Prostate Cancer Cells (PC-3)
Source: Molecules. 2023 Apr 4;28(7):3214. doi: 10.3390/molecules28073214 (PMC10096378; doi:10.3390/molecules28073214)
Supplement: Supplementary file 1 [file molecules-28-03214-s001.zip › molecules-2060326-supplementary.pdf]

Supplementary materials

The HPLC analysis of GMG:

A single peak of the compound can be seen from the HPLC chromatogram (Figure S1) at 0-5 minutes retention time compared with the crude extract chromatogram (Figure S2) which exhibited nine peaks and suggests an extract with different phytochemicals. The compound was further confirmed with NMR and LCMS analysis.

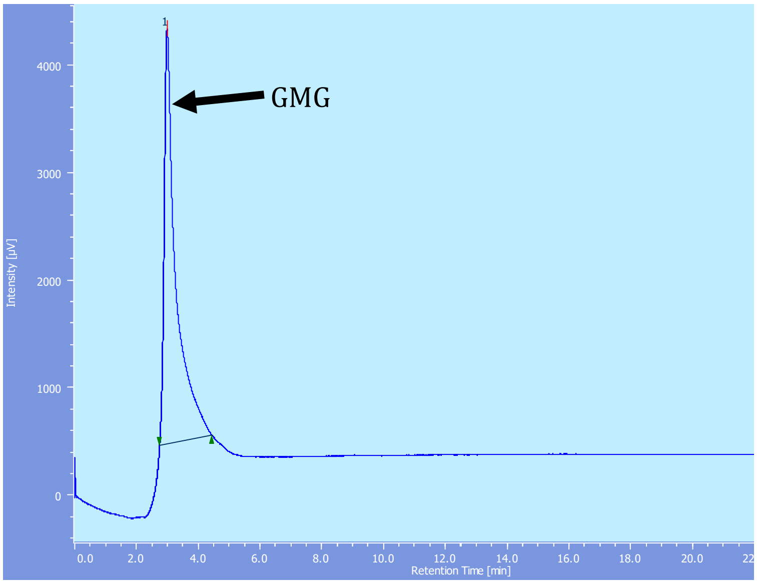

Figure S1. HPLC chromatogram of glucomoringin

The HPLC analysis of GMG, exhibited the presence of the compound. The peak represents the concentration of the compound.

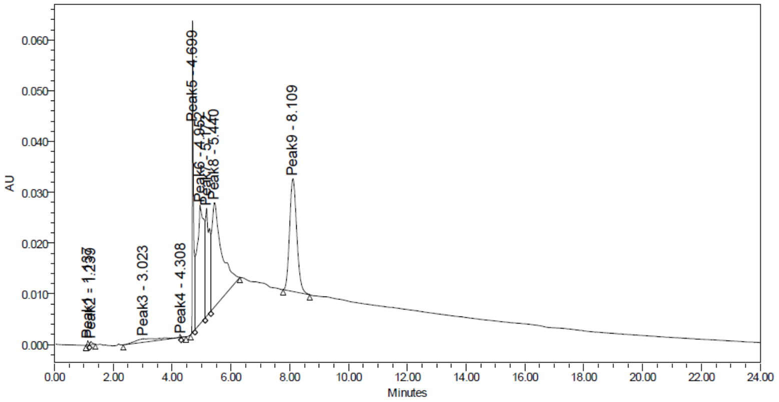

Figure S2. HPLC chromatogram of crude water extract of *M. oleifera* seed

NMR analysis (proton and carbon) data of Glucomoringin:

Analysis of 1H NMR spectral data shows the presence of a doublet deshielded at 4.74 and multiplet at 2.95 to 3.33, suggesting the presence of glucose moiety. The presence of aromatic compound was suggested to be deshielded at 7 and 7.21 with doublet, and 4.6 with singlet. The doublet was also deshielded, and suggests a rhamnose moiety which was at 4.55, 3.85 and 1.16. Multiplet and triplet were detected at 3.68 and 3.3 respectively and at 5.4 was identified as a broad singlet.

Table S1. NMR data of glucomoringin

GMG in D_20 (500/125 MHz)			GMG in D_20 (400/100 MHz) literature (De graaf et al., 2015)	
Carbon position	δ_H (in ppm, J in Hz)	δ_c (in ppm)	δ_H (in ppm, J in Hz)	δ_c (in ppm)
Glucose moeity				
1’	4.74, d (5)	81.58	4.57, d, (9.0)	80.9
2’	3.26-3.33 m	71.75	3.17–3.30, m	71.9
3’	3.26-3.33 m	76.92	3.17–3.30, m	77.0
4’	3.26-3.33 m	68.99	3.17–3.30, m	68.8
5’	2.95 m	76.92	3.09, m	79.7
6’	3.5 m	60.42	3.50, m	60.2
Benzylic moiety				
1		163.24		154.4
2	7.0, d, 10 Hz	118.17	7.00, d, (8.7)	117.4
3	7.21, d, 10 Hz	128.67	7.18, d, (8.7)	129.3
4				130.4
5	7.21, d, 10 Hz	128.67	7.18, d, (8.7)	129.3
6	7.0, d, 10 Hz	118.17	7.00, d, (8.7)	117.4
7	4.67, s,	35.87	3.86, dd (17.0)	37.2
Others		153.23		154.6
0				
Rhamnose moeity				
1”	5.4, s (broad)	99.69	5.41, d, (1.7)	98.1

2”	4.55, d, 10 Hz	68.99	4.03, dd, (1.7, 3.5)	69.9
3”	3.85, dd, 5, 5 Hz	-	3.86, dd, (3.5, 7.0)	70.0
4”	3.3, t, 10 Hz	71.75	3.38, t, (9.7)	70.2
5”	3.68, M	68.99	3.67, m	69.3
6”	1.16, d, 5 Hz	17.7	1.09, d, (6.3)	16.6

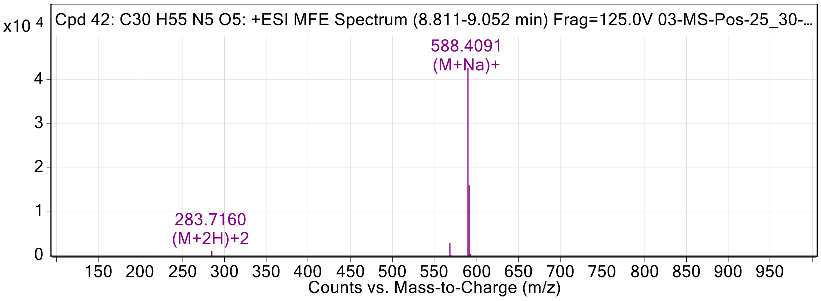

Figure S3. Mass spectrogram (ESI positive mode) of isolated glucomoringin

The mass spectrogram (Figure S3) confirmed the weight of the compound (588 m/z), thus clarifies that the compound was successfully isolated from the extract.
